# UAV-based multispectral image analysis revealed stay-green haplotypes in wheat specific for different soil nitrogen levels

**DOI:** 10.1186/s12870-025-07441-6

**Published:** 2025-10-21

**Authors:** Helen Behn, Agim Ballvora, Juliane Bendig, Facundo R. Ispizua Yamati, Ahossi Patrice Koua, Anne-Katrin Mahlein, Annaliese S. Mason, Uwe Rascher, Mohammad Bahman Sadeqi, Jens Léon

**Affiliations:** 1 Plant Breeding Department, Institute of Crop Science and Resource Conservation, University of Bonn, Kirschallee 1, Bonn, 53115 Germany; 2 Faculty of Agricultural, Nutritional and Engineering Sciences, University of Bonn, Campus Klein-Altendorf, Klein-Altendorf 2, Rheinbach, 53359 Germany; 3 Institute of Sugar Beet Research (IfZ), Holtenser Landstraße 77, Göttingen, 37079 Germany; 4 Institute of Bio- and Geosciences, IBG-2: Plant Sciences, Forschungszentrum Jülich GmbH, Leo-Brandt-Straße, Jülich, 52425 Germany; 5 Institute of Crop Science and Resource Conservation, Faculty of Agricultural, Nutritional and Engineering Sciences, University of Bonn, Bonn, 53115 Germany

**Keywords:** Stay-green, Senescence, Genome-wide association study, Haplotypes, Nitrogen use efficiency, Remote sensing, Multispectral image analyses, Plant senescence reflectance index, Wheat breeding

## Abstract

**Background:**

The so-called stay-green trait, a delay in onset and progression of leaf senescence, is associated with slower chlorophyll degradation and higher photosynthesis rates during maturation resulting in higher crop yields. Understanding the genetic and physiological basis of the stay-green trait and breeding cultivars with stable stay-green behaviour across a range of different nitrogen (N) conditions and specifically under low N availability can contribute to ensuring wheat yields and reducing N fertilizer application.

The goal of this study was therefore to identify haplotypes associated with high stay-green capacity under different N availability conditions in wheat. A diverse set of 221 wheat cultivars was grown under three different N levels and phenotyped by uncrewed aerial vehicle (UAV)-based multispectral imaging to characterise genetic and environmental variation in stay-green. Haplotypes associated with stay-green were identified across N levels and specifically under low N availability.

**Results:**

The plant senescence reflectance index (PSRI) calculated from multispectral images was identified as the most specific stay-green indicator allowing for differentiation of genotypic effects due to its greater sensitivity to senescence-related changes in pigment composition and its higher reliability. We found genetic variance for stay-green and a consistent genetic correlation between stay-green and grain yield at all imaging dates and N levels within the utilised diversity panel confirming its potential as a future breeding target.

Haplotype analyses revealed two favourable major allele haplotypes present in 95% of the stay-green cultivars, i.e. the top 25% of the diversity set based on PSRI values, which significantly enhance stay-green performance and grain yield. In addition, we identified a favourable minor allele haplotype specifically associated with stay-green under low N availability and capable of further increasing stay-green and grain yield when stacked onto the two favourable major allele haplotypes.

**Conclusions:**

The newly identified stay-green haplotypes can be further used for fine-mapping and identifying the underlying genes as well as for selecting for higher stay-green and grain yield. Thereby our results can contribute to improving our understanding of the complex genetic regulation underlying stay-green in different environments and to breeding new cultivars with stable performance across N levels or specifically under low N availability.

**Supplementary Information:**

The online version contains supplementary material available at 10.1186/s12870-025-07441-6.

## Background

Wheat (mainly bread wheat, *Triticum aestivum*, but also durum wheat, *T. turgidum*) has a major role in ensuring food security, accounting for around 27% of the average minimum daily energy requirement per person and for a comparable fraction of the recommended daily protein intake worldwide [1, 2]. It also has a high environmental impact, being the most-produced crop in terms of harvested area according to a ranking from 2022 [3].

The demand for staple crops such as wheat is increasing due to a growing world population [4]. At the same time biotic and abiotic threats to yield and yield stability, e.g. climate change, pathogens and pests [5, 6], are slowing down or ending the positive trend in wheat yield development [7, 8]. Different constraints to expanding the area of arable land, e.g. soil degradation or competition with biofuels [9], further aggravate this situation. Therefore, in spite of significant progress in improving wheat yield potential over the past seven decades, the requirement for wheat cultivars with higher and more stable yields still remains high [10, 11].

Several studies have shown that prolonging photosynthetic activity and maintaining its efficiency during the critical period of grain filling is an effective approach to increasing and stabilising grain yields of cereals [12, 13]. This so-called stay-green trait, a heritable delay in onset and progression of leaf senescence, is associated with slower chlorophyll degradation and nutrient remobilisation and with a higher photosynthesis rate during maturation [14–16]. The trait is considered functional if prolonged leaf greenness results in maintenance of photosynthetic activity, increased translocation of assimilates to harvested tissues, and ultimately higher yields. It is referred to as non-functional, if these processes are disrupted [12, 16]. The high correlation consistently found between stay-green and yield indicates that selection for functional stay-green could contribute to the development of adaptive high-yielding cultivars for diverse environmental conditions [17, 18]. The genetic variance for the stay-green trait within the wheat breeding germplasm pool underpins its high potential as a future breeding target [18–20].

Leaf senescence is closely related to its water and nitrogen status [21–23]. Under terminal water deficit, onset and progression of senescence were found to result from the source-sink balance between N supply and N demand [23, 24]. N demand is largely determined by grain number during maturation [23, 24]. N supply is fuelled by uptake of soil available N or senescence-related degradation and remobilisation from leaves, shoots and roots [23–25] Genotypic differences in stay-green can therefore be partly explained by differences in N uptake and utilisation efficiency [23–25]. N uptake efficiency defined as plant N per soil available N and N utilisation efficiency calculated as grain yield per plant N are key components of nitrogen use efficiency (NUE, defined as yield per soil available N) [26]. Therefore, a high NUE can make stay-green more independent of N supply [27]. In low-input agricultural systems, such as in parts of the global south, a high stay-green capacity based on high NUE can contribute to ensuring yields [28]. In high-input production systems as they are common in western Europe, a high stay-green capacity based on NUE will allow for a reduction in N fertilisation [29]. Reducing N fertilisation will help to minimise the environmental impact of wheat production and to achieve the objectives of the European Farm to Fork Strategy aiming to reduce fertiliser use by at least 20% by 2030 [30].

During plant senescence a series of profound biochemical and biophysical changes take place, altering the reflectance properties and the phenotype of the leaves. Repeated visual scoring of these phenotypic changes to determine onset and rate of the process is labour-intensive, highly subjective and mainly captures visible pigment changes while disregarding other characteristics [31]. In recent years, the visual assessment has therefore been replaced by non-destructive measurement methods of reflectance properties based on RGB (Red, Green, Blue), multispectral and hyperspectral sensors. These can be integrated in hand-held devices or mounted on uncrewed aerial vehicles (UAVs) to achieve the required throughput for phenotyping large diversity panels in diverse environments at high temporal resolution [32].

Various approaches have been proposed to derive senescence metrics from these data and characterise cultivar-specific senescence profiles. Some researchers have suggested calculating singular metrics such as relative senescence scores, relative stay-green scores, or elapsed thermal time to a particular senescence score [20, 33]. Other approaches include modelling such as using two-parameter or four-parameter logistic-type models or splines [34, 57] to derive characteristic turning points and periods such as the beginning, end or duration of senescence [31, 33] or to derive senescence metrics such as senescence rate or area under the curve [20, 31, 33]. Vegetation indices and full-spectrum models differ considerably in their sensitivity to senescence-specific spectral changes in leaf reflectance [31]. The PSRI for example has shown a high sensitivity. It captures primarily the chlorophyll/carotenoid ratio, which is subject to major changes during senescence [35]. In contrast, the Normalised Difference Vegetation Index (NDVI) was found to be relatively insensitive to physiological changes occurring at the leaf scale in the early phase of senescence [36].

In recent years, these advances in UAV-based high-throughput phenotyping of stay-green have been used for genome-wide association studies (GWAS) in crops [37, 38, 57]. The number of marker-trait associations (MTAs) identified in these studies and their dependence on the diversity panel and the environment support the understanding that stay-green is under complex genetic control [18–20, 37]. Gene ontology enrichment analyses based on marker-trait associations identified for stay-green in wheat suggested the significant association of the candidate genes with biological processes including leaf senescence, ethylene response, ageing and programmed cell death, and functions primarily related to nutrient reservoir activity [20]. These studies have revealed various physiological mechanisms underlying the senescence process and its regulation. However, the genetic basis of stay-green behaviour in wheat is still poorly understood [20].

To further elucidate the genetic basis of stay-green in wheat and its relationship with N availability, we conducted a two-year field experiment with 221 wheat cultivars grown under three different soil N levels. Stay-green was characterised using UAV-based multispectral imaging for subsequent use in GWAS and identification of genetic regions underlying this trait. The goals of the present study were (1) to compare vegetation indices from multispectral drone images and identify the most specific, sensitive and reliable stay-green indicator, (2) to characterise genetic and environmental variance for stay-green in the diversity panel at different time points, (3) to identify favourable haplotypes for genetic markers associated with stay-green across nitrogen fertilisation levels and specifically under low N availability, and to assess their effects on stay-green and grain yield, and (4) to examine allele frequencies in the present diversity panel for introduction and indirect selection of favourable stay-green alleles over the past decades.

## Methods

### Plant material

The study was performed using a diversity set comprising 221 bread wheat cultivars. These included 165 German cultivars and 56 accessions from other European countries, USA, Mexico, India and Australia representing the global genetic diversity. The German cultivars covered 50 years of German bread wheat breeding history from 1966 to 2016 and all baking quality classes. They were selected based on their economic and agronomic importance in German wheat production during their period of release. Details on cultivars including year and country of registration are provided in Tab. S1. The composition of the utilised diversity panel has been described before [39, 44].

### Single nucleotide polymorphism genotyping

Genotyping of the accessions of the diversity set was carried out at SGS Trait-Genetics GmbH (Gatersleben, Germany) using the Infinium iSelect 15 K single nucleotide polymorphism (SNP) bead array comprising 13 006 polymorphic loci [40], as well as the 135 K Axiom exome capture array carrying 136 780 SNP markers [41]. The resulting SNP data were merged in a single dataset and aligned to the reference wheat genome assembly RefSeq v1.0 [42] using the alignment tool Bowtie2 version 2.3.4.3 [43]. Filtering and quality control procedures were applied to the markers as previously described [44] to select for high-quality locus-specific SNPs which were accurately mapped to the reference genome. Monomorphic markers and SNPs with minor allele frequencies (MAFs) below 3% and/or with more than 5% missing data were discarded. Ultimately, 24 216 informative SNP markers (Tab. S2) with defined physical positions were retained for further analysis [44]. The SNP dataset utilised in this study has previously been published and is freely accessible (supplementary table S2 in [44]).

### Multi-year, multi-environment field experiments

**Fig. 1:**
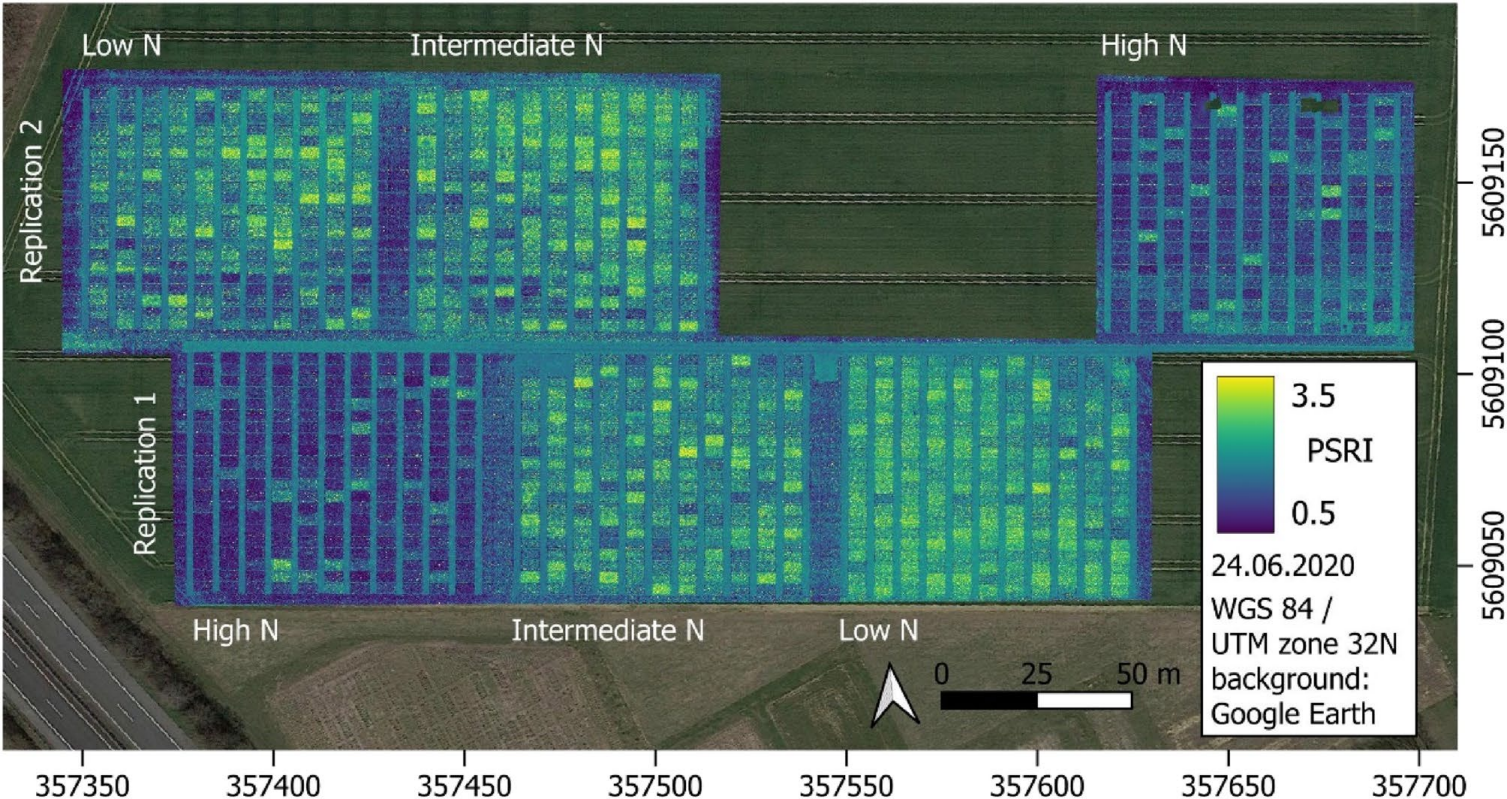
Overview of the field experiment at Campus Klein-Altendorf, research facility of the University of Bonn. The experiment was designed as a split-plot design with three N levels (high, intermediate and low N) as main plots, two blocks (replications) within each main plot and the complete set of cultivars (221) in each of the blocks. The experimental area is shown as Plant Senescence Reflectance Index calculated from UAV-based multispectral images recorded on 24 June 2020. Unit of axis scaling: metres

Field experiments were conducted at Campus Klein-Altendorf, a field research facility of the University of Bonn (geographic location: 50.61^°^N, 6.99^°^E, and 187 m above mean sea level), under natural field conditions in two consecutive growing seasons from 2018 to 2019 and from 2019 to 2020. The annual trials were conducted in a split-plot design with three nitrogen fertilisation levels as the main plots. Within these main plots, the experimental design was a fully randomised block layout with 221 cultivars in each of the two blocks (replications, see Fig. 1). Technically, the 221 cultivars were sown in 21 columns and 11 rows, creating additional plots which were used for seed propagation. The three nitrogen fertilisation levels were high *N* = 220 kg N/ha (including mineral soil nitrogen, N_min_), intermediate *N* = 110 kg N/ha (including N_min_), and low N = no additional N fertiliser (only N_min_). These are hereafter referred to as low, intermediate and high N level. Fertilisation doses were adjusted to N_min_ by first determining N_min_, then defining N_min_ as the low nitrogen level, and finally dosing the main plots with intermediate and high N content up to the target concentrations of 110 and 220 kg N/ha, respectively. The average N_min_ value was determined according to standard methods [45, 46] as described in a previous study [47] from a mixed sample obtained from a total of 18 sampling points arranged in a diagonal scheme across the experimental area. In 2019 N_min_ was 93 kg N/ha; in 2020 it was 81 kg N/ha. The intermediate N level was achieved by a single fertiliser application given in February 2019 and March 2020, respectively. The high N level was achieved by three fertilisation applications in February, April, and June 2019 and in March, April, and June 2020, respectively. The position of the N fertilisation levels within the experimental area was completely randomised each year. The whole experimental area was changed annually in a crop rotation system.

Wheat cultivars were grouped according to their height and maturation to reduce neighbour effects due to differences in plant size and maturity. Grouping was based on prior examination of the diversity panel. Within these groups cultivars were completely randomised. The wheat cultivars were sown at a density of 330 seeds per square metre in 7 × 3 m sub-plots with a harvesting area of 5 × 1.65 m in the centre of the area (plot in plot design).

Fertilisers, except for nitrogen, were applied according to requirements determined individually each year to prevent confounding effects from basic nutrient deficiencies. Fungicide application and weed control followed standard management procedures recommended for the region.

### Growing degree days

Cumulative growing degree days (GDDs) were calculated according to the Peterson equation as the sums of the averages of the daily temperature minimum and maximum minus the basic threshold temperature (°C) [48]. A basic threshold temperature of 4.0 °C [48] and an upper limit of 25 °C were defined [49]. Values below the lower or above the upper limit were set to these threshold values. Cumulative GDDs were calculated for the time periods from January 1^rst^ to the respective imaging dates [48]. Imaging dates were excluded from the addition because flights were carried out in the mornings.

Meteorological data were collected by a local weather station (from GWU-Umwelttechnik GmbH, Talstr. 3, 50374 Erftstadt, geographic location: 50.37°N, 6.59°E, 180 m above mean sea level). Worldwide the year 2020 was the second warmest on record. In Germany, the average temperature (10.4 °C) was 2.2 degrees above the reference period of 1961 to 1990 and total precipitation per year (710 l/m²) was around 10% below the average (789 l/m²) [50]. Yet, there was no exceptional heat period and rainfall was regular throughout the entire data collection phase.

### Phenotypic data collection

Plant height was measured manually. Grain and straw yield were determined from the combined harvest of the whole plots. Harvest index was calculated as the grain yield divided by total above-ground biomass (i.e. straw yield plus grain yield) in kg/plot. Grain yield per plot was determined by threshing from the mature standing canopy. Immediately after threshing, grain moisture was measured and grain yield was corrected to a standard moisture of 14%. A random grain subsample from each plot was used to determine the thousand-kernel weight. Nitrogen use efficiency was calculated for each cultivar and N fertilisation level by dividing cultivar-specific grain yield in g/m^2^ by soil available nitrogen per square metre [51]. Relative leaf chlorophyll content was estimated non-destructively using a chlorophyll metre (SPAD-502, Konica Minolta Sensing Europe B.V., Nieuwegein, Netherlands). Measurements were performed on the flag leaf from the main tiller of three representative plants of each cultivar per replication. The average of these six plants per cultivar and treatment level was used as an estimate of the chlorophyll content. All measurements were taken between 11 am and 3 pm on 27 May 2020 (777 GDDs, early heading stage).

### Multispectral tracking of senescence

#### Image acquisition and processing

Multispectral image data were collected with a MicaSense RedEdge Dual camera system in 2020 and with a RedEdge-MX camera in the reference year 2019 (AgEagle Aerial Systems Inc., Wichita, KA, USA). Both were equipped with a downwelling light sensor (DLS). The MicaSense RedEdge Dual camera system was used to capture multispectral images, recording a total of 10 spectral bands covering the range from blue to near-infrared radiation. The bands, their respective centre wavelengths and bandwidths are as follows: coastal blue (444 nm, 28 nm), blue (475 nm, 32 nm), green (531 nm, 14 nm), another green (560 nm, 27 nm), red (650 nm, 16 nm), another red (668 nm, 14 nm), red edge (705 nm, 10 nm), another red edge (717 nm, 12 nm), near-infrared (740 nm, 18 nm), and an additional near-infrared (842 nm, 57 nm). Of these, the bands at 475, 560, 668, 717, and 842 nm are also captured by the MicaSense RedEdge-MX system, which contains only one of the two cameras operating in the dual system. The camera system was mounted on a multirotor UAV, Matrice 600 Pro (DJI, Shenzhen, China). The UAV was flown at 30 m above ground level, with a front overlap of 90% and side overlap of 70%, resulting in a pixel size of 2 × 2 cm. Two flights were carried out at each time point to cover the entire field. A set of eight or nine Lambertian reference panels of different grey levels [52] was placed next to the experiment for each flight and used for calculating top of canopy reflectance. Flights were conducted within two hours of local solar noon under clear sky (27 May and 24 June 2020 and 7 June 2019) or homogeneous overcast (9 June and 7 July 2020) conditions. One imaging date (27 May 2020/777 GDDs) was just before the average heading date on 2 June 2020 with a standard deviation (SD) of 3.7 days. Three imaging dates (9 June/907 GDDs, 24 June/1107 GDDs and 7 July 2020/1290 GDDs) were between heading and the average yellow maturity date on 17 July 2020 (SD = 2.9 days). Heading is considered a visible indicator of subsequent flowering which is more difficult to determine in self-pollinating plants with closed flowers. Hence, the imaging dates 9 and 24 June 2020 lie within the period of flowering and grain filling which is also prone to the onset of senescence. At the last imaging date (7 July 2020) most plants were already in an advanced stage of senescence, and hence data from this flight were excluded from the repeated-measures analysis of variance (ANOVA) and the GWAS.

Images were processed in *Metashape Professional* (version 1.8.1 build 13915) by Agisoft [53]. Image alignment was done at high accuracy and further optimised using 19 ground control points with known geographic coordinates as a reference. Orthomosaics were constructed via an intermediate high accuracy dense cloud and exported in TIF format at a resolution of 2 × 2 cm. Calibration of orthomosaics was done according to the empirical line reflectance calibration method described by Chakhvashvili et al. [52] using *QGIS* software (Desktop version 3.28) [54].

#### Calculation of vegetation indices and choice of stay-green indicator

Various vegetation indices (VI) were calculated from the spectral bands of the orthomosaics as detailed in Table 1. These included the classical chlorophyll and biomass index NDVI [20] as well as indices that have been more specifically used to track senescence in recent studies such as the Normalised Difference Red Edge Index, NDRE [57], and PSRI [31]. Calculations were carried out in *R* software (version 4.4.0 for Windows) [58]. Formulas and spectral bands used to calculate VI are given in Table 1. Since the PSRI was originally proposed as (678 nm–500 nm)/750nm [35], we used the 740 ± 9 nm band as near-infrared (near-IR) band in the main dataset from the harvest year 2020. In the reference dataset from 2019 lacking this spectral band we used the 842 ± 29 nm band as near-IR band instead. However, moving the near-IR band towards higher wavelengths was reported to have little effect on the accuracy of the index [31].

Medians per field plot of the calculated vegetation indices were extracted in R using the *terra* package (version 1.7.71) [59] and shape files previously generated in QGIS. These covered the central area of each plot with a uniform size of 6.4 m². In the 2020 median dataset five field plots were excluded from the following analyses due to missing or extremely poor vegetation coverage.

The relationship between the calculated vegetation indices and leaf chlorophyll content (SPAD) was estimated by means of a Pearson correlation coefficient across and within N levels (measurement date: 27 May 2020). Likewise, the vegetation index selected as indicator for estimating the degree of senescence (PSRI) was examined for correlation with growth and yield-related traits.

Reliability of phenotypic assessment of the cultivars by the vegetation indices was estimated according to Bernardo [60] using the following formula [61].

$$\begin{aligned}\text{Reliability}=&\text{V}_\text{C}/\left(\text{V}_\text{C}+\left(\text{V}_{\text{C}\times \text{N}}/\text{n}\right)\right. \\& \left.+\left(\text{V}_\text{R}/\text{r}\times \text{n}\right)\right)\end{aligned}$$

where V_C_ is the variance component due to the cultivars, V_CxN_ represents the variance component due to the interaction between cultivars and N levels, V_R_ is the residual variance, n stands for the number of N levels, and r is the number of replications (number of blocks × number of replicates per block). The variance components estimated as best linear unbiased estimators (BLUEs) have been calculated based on the linear mixed model given below by setting cultivar and cultivar-by-N level interaction as random factors. The standard error of reliability was calculated using the method by J. Holland [62].

Based on its sensitivity for senescence-related changes in leaf reflectance and its reliability, the PSRI was chosen as stay-green indicator for subsequent analyses. The PSRI increases with increasing degree of senescence. This means the PSRI is inversely correlated with stay-green and low PSRI values indicate a high degree of stay-green, whereas high PSRI values are associated with a low degree of stay-green. Accordingly, allelic variants referred to as `favourable´ alleles are associated with lower PSRI values, whereas those designated as `unfavourable´ are associated with higher PSRI values. The highest performing 25% of cultivars based on PSRI values during the central senescence phase (9 and 24 June 2020) are referred to as `stay-green cultivars´ in the following.

**Table 1:** Calculated vegetation indices with formulas, centre wavelengths of the utilised spectral bands, and references of their previous use as stay-green indicators

Vegetation index	Formula and spectral bands
Normalised Difference Vegetation Index (NDVI) [19, 20, 37, 55, 56]	(Near-IR - Red)/(Near-IR + Red)
	(842 nm–668 nm)/(842 nm + 668 nm)
Normalised Difference Red Edge Index (NDRE) [20, 56, 57]	(Near-IR - Red Edge)/(Near-IR + Red Edge)
	(842 nm–717 nm)/(842 nm + 717 nm)
Plant Senescence Reflectance Index (PSRI) [31]	(Red/Blue) - Near-IR
	(668 nm/475 nm) − 740 nm (2020 experiment) (668 nm/475 nm) − 842 nm (2019 experiment)

### Statistical analyses of selected stay-green indicator

#### Linear mixed model

Data were prepared by centring and scaling the explanatory variables growing degree days and N level. A square root transformation was applied to the selected vegetation index PSRI after adding a constant (10) to obtain exclusively positive values [63].

The following linear mixed model was fitted to the data

$$\begin{aligned}{\mathrm Y}_{\mathrm{ijkl}}=&\mathrm\mu+{\mathrm N}_{\mathrm i}+{\mathrm B}_{\mathrm j}+{\mathrm{nb}}_{\mathrm{ij}}+{\mathrm C}_{\mathrm k}\\&+{\mathrm{CN}}_{\mathrm{ki}}+{\mathrm{cnb}}_{\mathrm{kij}}+{\mathrm T}_{\mathrm l}\\&+{\mathrm{TN}}_{\mathrm{li}}+{\mathrm{TC}}_{\mathrm{lk}}+{\mathrm{TNC}}_{\mathrm{lik}}\\&+{\mathrm\varepsilon}_{\mathrm{ijkl}}\end{aligned}$$

where Y_ijkl_ represents a measured value of the dependent variable PSRI in the respective sub-plot, µ is the grand mean, N_i_ represents the fixed effect of nitrogen fertilisation level i, B_j_ is the fixed effect of replication j, C_k_ stands for the fixed effect of cultivar k, T_l_ is the fixed effect of time point of data collection l in units of GDDs. The remaining model terms represent the respective interaction effects and three error terms, a main plot error (bn_ji_), a sub-plot error (bnc_jik_), and a residual error (ε_ijkl_). Hence, the model has the structure of a split-plot design [64, 65] or a repeated measures design [66] accounting for the dependence and covariance between repeated measurements. This is achieved by incorporating the sub-plot error, a random variable for the smallest experimental units, and thereby a compound symmetry structure. Error terms are denoted by lower case letters to distinguish these random effects from fixed effects.

Genotypic effects for GWAS were modelled as random effects (BLUEs, Tab. S3). The effect of block was set as fixed in the model as it only has two factor levels. This follows the general rule that it requires five or more factor levels to benefit from setting a factor as random [67].

The model was fitted using the *mixed* function of the *afex* software package (version 1.4-1) [68], which estimates mixed models with the *lmer* function of the *lme4* package [69] and calculates p-values for all fixed effects. The function then passes the model to the *anova* method from the *lmerTest* package [70] to run the mixed model ANOVA using Type III sums of squares.

P-values were calculated using the Kenward-Roger method, which provides a high protection against anti-conservative results, and subjected to a Greenhouse-Geisser correction. The Greenhouse-Geisser procedure is commonly applied to correct p-values if the sphericity assumption of a repeated measures ANOVA is not met based on Mauchly’s test. Sphericity in this case refers to the equality of the variances of the differences between scores for any two time points.

The main data set from the harvest year 2020 was compared to the reference year 2019. To estimate the strength of the relationship between the stay-green performances of individual cultivars in the two years, a Pearson correlation between genotypes BLUEs values for PSRI measured on 9 June 2020 (907 GDDs) and 7 June 2019 (808 GDDs) was calculated. However, in 2019 a MicaSense RedEdge-MX camera capturing only five spectral bands was used instead of the dual camera system used in 2020. Therefore, from the 2019 image dataset PSRI was calculated using a different NIR band (842 ± 29 nm instead of 740 ± 9 nm).

#### Calculation of relative senescence rate

Relative senescence rates (RSR) were calculated according to a formula suggested by Yu [20] which was adapted to PSRI:

$$\begin{aligned}\text{RSR}=&((\text{PSRI}_{\text{later date}}-\text{PSRI}_{\text{earlier date}})/\text{PSRI}_{\text{earlier date}})\\&\times100\end{aligned}$$

where PSRI_later date_ is the PSRI value at the later date and PSRI_earlier date_ is the PSRI value at the earlier date of a given time period. This results in the percent change in PSRI between the earlier and the later date. The modification was necessary because in contrast to most other VI, the PSRI is inversely proportional to plant greenness. RSRs were calculated for the time periods from 27 May to 9 June 2020 (777 to 907 GDDs) and from 9 June to 24 June 2020 (907 to 1107 GDDs).

### Genome-wide association study

A genome-wide analysis of marker-trait associations between PSRI BLUEs and all 24 216 SNP markers (supplementary table S2 in [44]) was performed using the *Genome Association and Prediction Integrated Tool (GAPIT) for R* software (version 3) [71]. The analyses were run for all three imaging dates across N levels and separately for the low N level. Two multi-locus models were used for GWAS, the Bayesian-information and Linkage-disequilibrium Iteratively Nested Keyway (BLINK) and the Fixed and random model Circulating Probability Unification (FarmCPU) [72]– [73]. Both models use the first three principal components derived from all markers as covariates to reduce false positives due to population stratification. In addition, BLINK iteratively incorporates associated markers as covariates for testing markers to eliminate their connection to the cryptic relationship among individuals. These associated markers are selected based on linkage disequilibrium (LD) and optimised for Bayesian information content and re-examined across multiple tests to reduce false negatives.

To account for multiple testing and avoid false positive MTAs, the significance level was adjusted using the Bonferroni correction (shown as a green line in the Manhattan plots). In addition, p-values calculated for marker-trait associations were corrected using the Benjamini-Hochberg procedure and a false discovery rate threshold cut-off (5%) was determined, which is indicated as a dashed green line in the Manhattan plots [74]. Principal component analysis was conducted in R using the *prcomp* function from the *stats* package [75].

### Identification of stay-green haplotypes

The haplotypes were identified by k-means analysis in *SAS* software version 9.4 *Proc fastclus* [76]. Adjacent markers with an LD greater than 0.7 to the marker of interest were included. At least five members were required to form a haplotype. Haplotype codes consist of a letter in the first position indicating whether it is a major or minor allele, a second letter for the respective deoxyribonucleic acid (DNA) base and optionally a letter indicating different variants. Haplotypes identified as corresponding to the major (most frequently observed) allele were labelled by the capital letter M. Haplotypes identified for the minor allele were coded as the lowercase letter m. DNA bases are abbreviated by their first letters as C = cytosine, T = thymine, G = guanine and A = adenine. If there were different variants of a major or minor allele haplotype, this was denoted by the letter v.

Significant differences between three or more haplotypes were determined using an ANOVA followed by a Tukey’s honest significant difference test, or in the case of unequal variances by a Kruskal-Wallis test followed by a Wilcoxon test. In case of comparisons between two haplotypes significant differences were assessed using a Student’s t-test for equal variances or a Welch’s t-test in case of unequal variances. Differences indicated as significant in the following were at least significant at the 5% level (α = 0.05). Explicitly specified significance levels were below α = 0.05.

## Results

### Vegetation indices significantly predict chlorophyll content

All three vegetation indices, NDVI, NDRE and PSRI, significantly predicted chlorophyll content (SPAD, p-values ≤ 0.001) in the regression analyses across N fertilisation levels and at each individual N level.

Across N levels, Pearson correlation with SPAD was moderate for NDVI (*r* = 0.57) and strong for NDRE (*r* = 0.64) and PSRI (*r* = −0.61, p-values < 2.2e-16, measurement date: 27 May 2020, Fig. 2). However, the correlation results for NDVI showed clear signs of saturation. Hence, PSRI and NDRE were selected as possible stay-green indicators for further analyses.

**Fig. 2:**
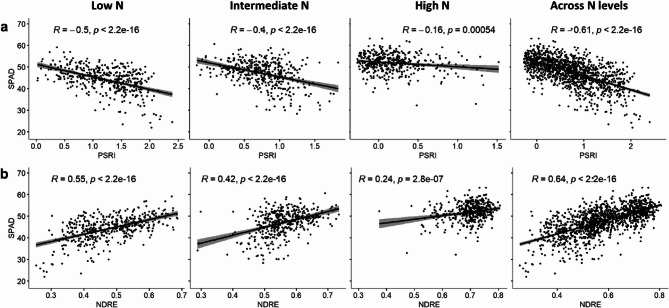
Scatter plots with Pearson correlations between (a) SPAD and PSRI and (b) SPAD and NDRE calculated for low N (81 kg/ha), intermediate N (110 kg/ha), high N (220 kg/ha), and across N levels. Sampling date: 27 May 2020 (777 GDDs). *R* Pearson correlation coefficient, *p* p-value. The outer edges of the shaded areas represent the 95% confidence intervals calculated for the correlations

Within the treatment groups, PSRI showed the strongest correlation with SPAD at low N (*r* = − 0.5) and weaker relationships at intermediate (*r* = − 0.4) and high N (*r* = − 0.16, Fig. 2). NDRE showed a similar pattern with the strongest relationship at low N (*r* = 0.55) and declining strengths at intermediate (*r* = 0.42) and high N (*r* = 0.24). All correlations were significant at least at the 0.1% level (*p* ≤ 0.001).

### Genetic and environmental variance in stay-green present at all imaging dates

**Fig. 3:**
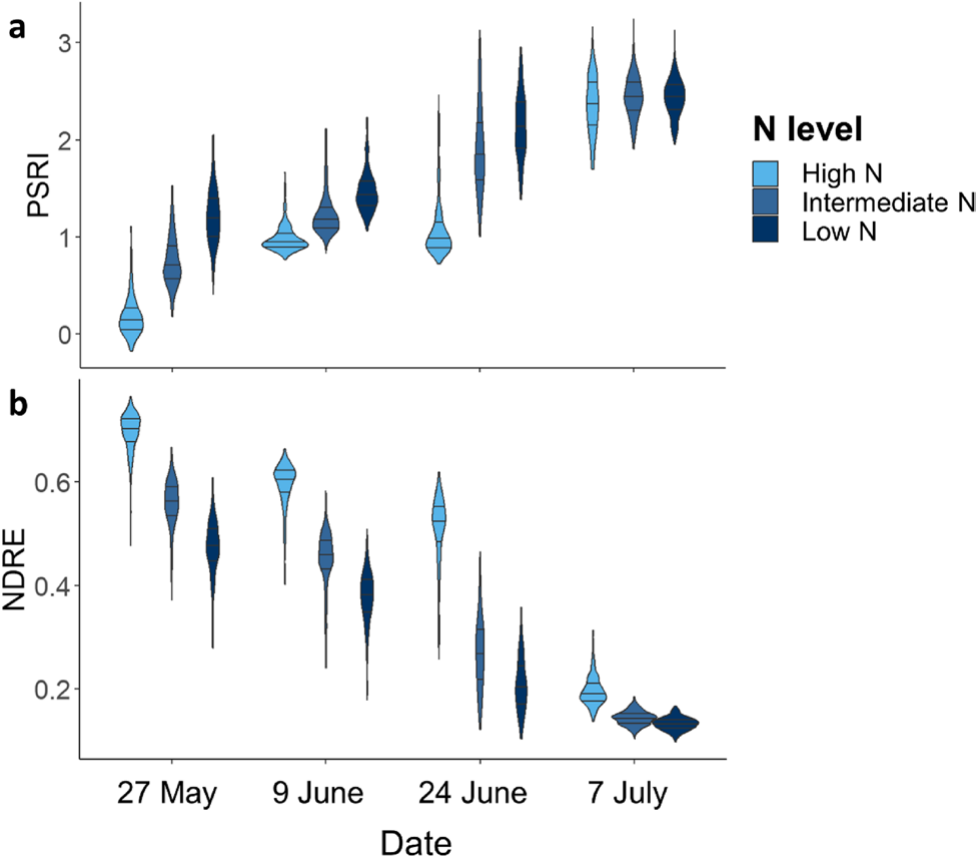
(**a**) PSRI and (**b**) NDRE by N level over time. Low *N* = 81 kg/ha, Intermediate *N* = 110 kg/ha and High *N* = 220 kg/ha. Dates translate to growing degree days as follows: 27 May = 777 GDDs, 9 June = 907 GDDs, 24 June = 1107 GDDs and 7 July 2020 = 1290 GDDs

**Fig. 4:**
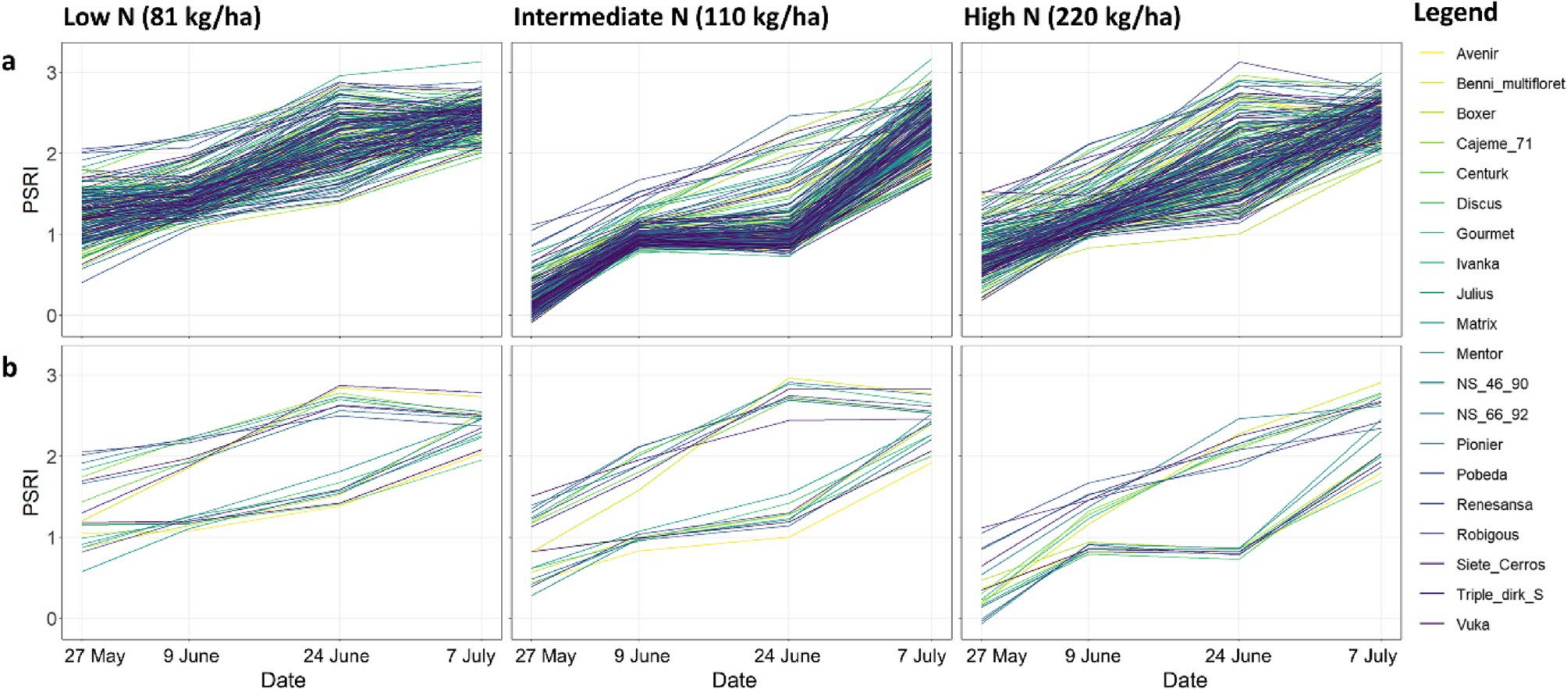
Time course of the stay-green indicator PSRI by N level (panels) and cultivar (colour coding as indicated in the legend) shown for (**a**) all cultivars and (**b**) the ten highest and lowest performing cultivars based on their genotypic effects on PSRI during the central senescence period (9 and 24 June 2020). The ten highest performing cultivars were Avenir, Pionier, Vuka, Discus, Boxer, Gourmet, Robigous, Matrix, Mentor and Julius. The ten cultivars with the poorest stay-green performance were NS-46-90, Pobeda, Cajeme 71, Ivanka, Renesansa, Siete Cerros, Triple dirk S, NS-66-92, Benni multifloret and Centurk. 27 May = 777 GDDs, 9 June = 907 GDDs, 24 June= 1107 GDDs, and 7 July 2020 = 1290 GDDs

Stay-green performance was estimated by the vegetation indices NDRE and PSRI. Genetic variance for stay-green was present at all three time points and N levels as shown by the mixed model ANOVA (Figs. 3 and 4, and Tab. S7). The greatest genetic variance was observed at an advanced stage of senescence (24 June 2020/1107 GDDs). Environmental variance for stay-green was also present at all imaging dates. Stay-green was consistently greater at high N, whereas senescence began earlier and progressed faster at low N. While the effects of cultivar and N level were significant, their interaction was not. However, there were significant interactions between N level and time as well as cultivar and time (*p* ≤ 0.001).

The stay-green performance of individual cultivars was highly correlated between the repetitions of the experiment. For PSRI BLUEs a high Pearson correlation was found between 9 June 2020 and the reference date 7 June 2019 (*r* = 0.64, p-value < 2.2e-16, Fig. S1).

### PSRI chosen as stay-green indicator due to higher reliability

**Fig. 5:**
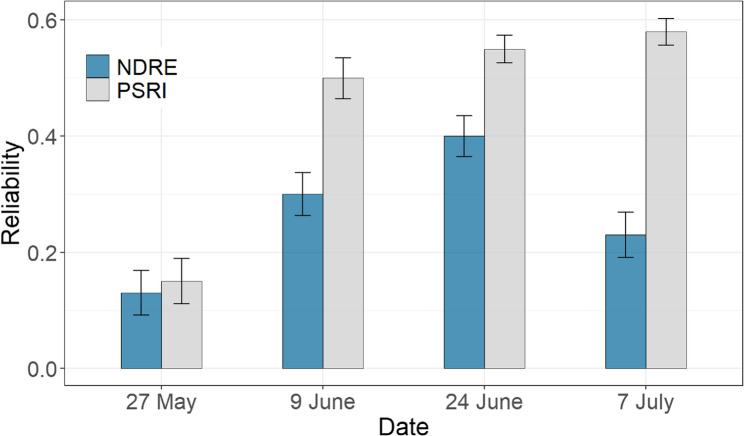
Reliability according to Bernardo [60] for PSRI and NDRE at the four imaging dates. 27 May = 777 GDDs, 9 June = 907 GDDs, 24 June = 1107 GDDs and 7 July 2020 = 1290 GDDs

Estimates of reliability calculated for NDRE and PSRI across N levels according to Bernardo [60] varied between the two vegetation indices and the imaging dates (Fig. 5). However, reliability was consistently higher for PSRI than for NDRE. PSRI was therefore chosen as stay-green indicator for the following analyses. This decision was supported by previous studies reporting a higher sensitivity of the PSRI in tracking senescence dynamics [31, 35].

### High performing cultivars show temporary decline in relative senescence rates

Senescence rates representing relative changes (%) in PSRI between two time points varied between cultivars, N levels, and time periods (Fig. 4, Tab. S4a + b). The greatest variability was observed between 9 and 24 June 2020, when part of the diversity set showed a decline in relative senescence rates whereas others continued to grow senescent at unaltered speed (Fig. 4a, Tab. S8). Comparing the ten highest with the ten lowest performing cultivars based on their genotypic effects on PSRI for each N level (Fig. 4b) shows that this slowdown in senescence was most pronounced at high N.

### Correlation between PSRI and yield strongest under low N availability

**Fig. 6:**
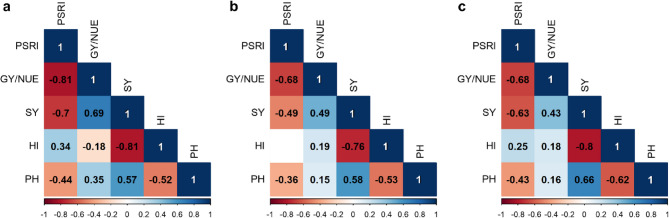
Pearson correlation for the stay-green indicator PSRI and phenotypic traits at (**a**) low, (**b**) intermediate and (**c**) high N, respectively. PH = plant height, GY/NUE = grain yield and nitrogen use efficiency (which share the same correlation coefficients), HI = harvest index, SY = straw yield. Results are shown for 9 June 2020 (907 GDDs). Colour code: shades of red = negative correlations, shades of blue = positive correlations, darker colours correspond to higher correlation coefficients and *vice versa*. All correlations indicated by a Pearson correlation coefficient and colour were significant at the 5% level

PSRI was found to be strongly correlated with grain yield and NUE across and within N levels (Fig. 6). Since NUE is calculated from grain yield, both share the same correlation coefficient. The genetic correlation between PSRI and grain yield/NUE increased throughout the course of senescence from − 0.49 (27 May) to −0.60 (9 June) and − 0.68 (24 June 2020). Within N levels, the correlation was highest at low N (*r* = −0.81) and lower at intermediate and high N (*r* = −0.68). PSRI also showed a high correlation with straw yield (ranging from − 0.7 to −0.49) and a moderate correlation with manually measured plant height (ranging from − 0.44 to −0.36, 9 June 2020/907 GDDs). The strongest correlations of PSRI with all four traits were found at low N.

### Diversity panel represents five decades of plant breeding history

**Fig. 7:**
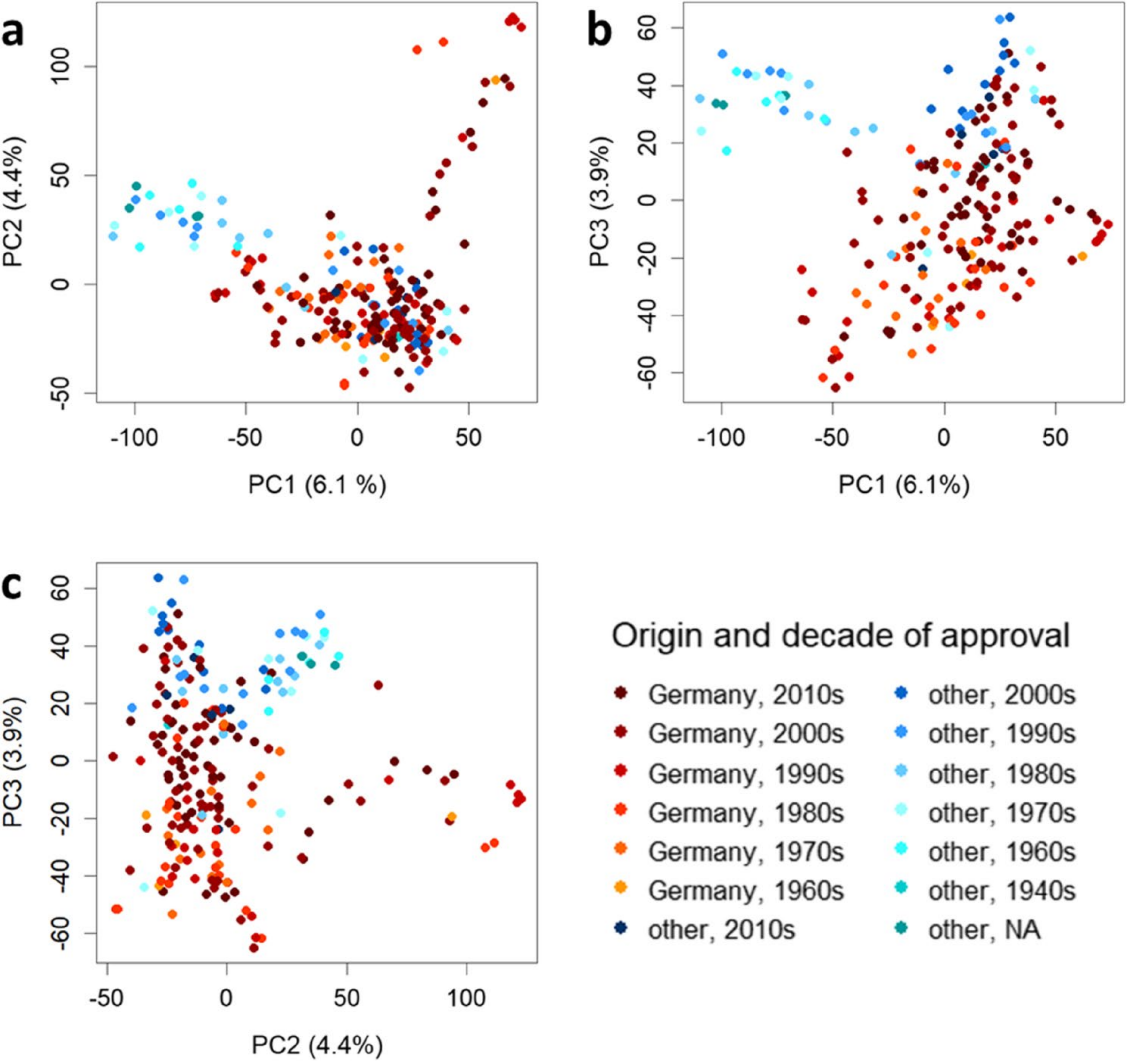
Principal components calculated based on all 24 216 SNP markers and coloured by origin group (German/other) and decade of registration: (**a**) PC1 and PC2, (**b**) PC1 and PC3, and (**c**) PC2 and PC3

The utilised diversity set of 221 cultivars represents the genetic diversity from five decades of plant breeding progress in Germany and other geographic regions as described previously [44]. For the principal component analysis, the cultivars were assigned to 14 groups based on origin (Germany/other) and decade of release. The plots showing principal components (PC) 1 to 3 indicate several overlapping clusters in the diversity panel (Fig. 7). Although no clear separation is apparent, some relevant patterns can be identified in the graphs. For example, the more recent German genotypes differ from the older genotypes from other origins in the first component (Fig. 7a + b). However, it is important to note that this component explains only 6.1% of the total genetic variance present in this cultivar set.

### Marker-trait associations for stay‑green identified across N levels and specifically at low N

In total, 26 unique marker-trait associations were identified, 16 across N levels and 11 under low N availability with one duplication (AX-111561744). Some of these Markers were repeatedly identified by both GWAS models (AX-111561744 and wsnp_BQ160404A_Ta_1_1) or in both years (AX-111561744 and AX-158618766). Others were identified at all three imaging dates in 2020 such as the locus on chromosome 7 A specified by the markers Kukri_rep_c68371_1242 and BS00061911_51.

**Fig. 8:**
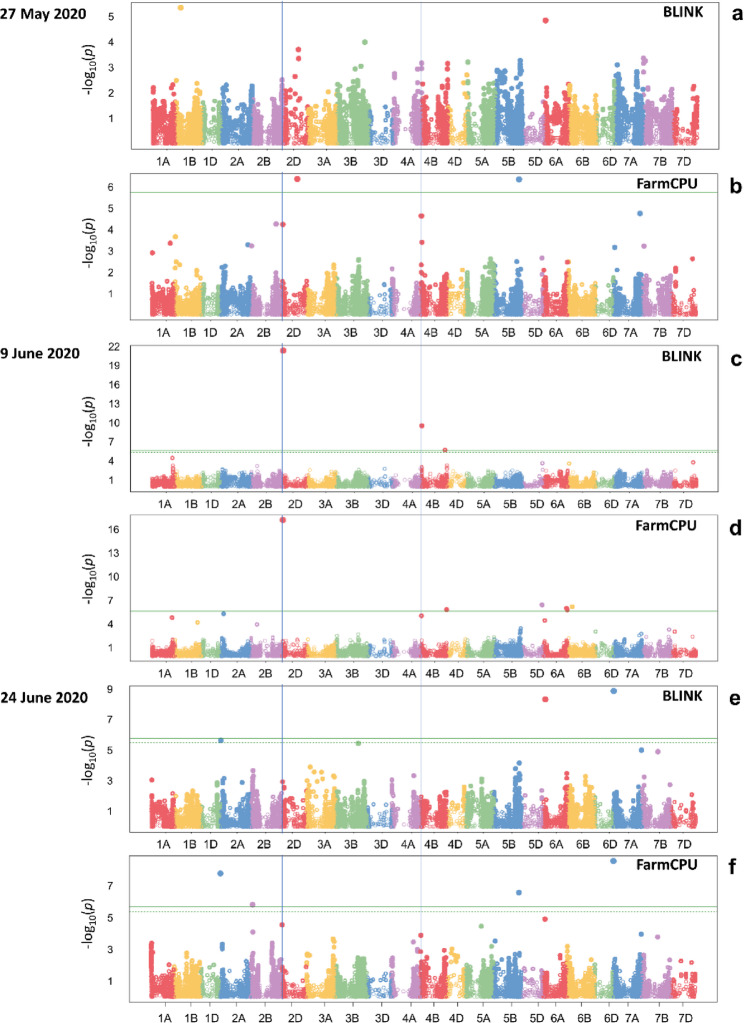
Manhattan plots showing significant marker-trait associations identified for the stay-green indicator PSRI across N levels on (**a**-**b**) 27 May = 777 GDDs, (**c**-**d**) 9 June = 907 GDDs, and (**e**-**f**) 24 June 2020 = 1107 GDDs by GWAS model (BLINK and FarmCPU). The green line indicates the Bonferroni multiple test threshold to determine significance of associations. The dashed green line shows the false discovery rate threshold cut-off (5%). The reported marker-trait associations for AX-111561744 on chromosome 2D and AX-158618766 on chromosome 4B are highlighted by blue vertical lines

Across N levels, three large effect loci with a proportion of variance explained (PVE) > 10% were identified (Table 2, Tab. S5 and Fig. 8). Among these large effect loci, the favourable major allele markers AX-111561744 and AX-158618766 explained the largest proportions of phenotypic variance in the central senescence phase (9 June 2020) accounting for 58.3% and 24.5%, respectively. Both MTAs have also been identified across N levels in the reference year 2019 (Table 2 and Tab. S5). One of the three large effect loci identified across N levels corresponds to a favourable minor allele (AX-111103882). However, although the marker AX-111103882 accounts for a PVE of 11%, its effect size is relatively small (0.005).

**Table 2:** Large effect loci (PVE > 10%) associated with the stay-green indicator PSRI across N levels and under low N availability in genome-wide association studies by imaging dates

Across *N* levels										
Date/ GDD	SNP (favourable allele)	Chr	Pos (Mbp)	Haplotype (Mbp)	MAF	B&H *p*-value	Effect	PVE	Model	Prev. identified
9.6.20 907	AX-111561744 (G)	2D	23.4	21.9–23.4	0.05	9.6E-18	−0.024	58.3	BLINK	PBW, NAB, NUEBio, NUpE, SDW, SPAD, YII [77] Found in 2019
9.6.20 907	AX-158618766 (C)	4B	15.3	14.3–15.4	0.07	3.3E-6	−0.014	24.5	BLINK	Found in 2019
9.6.20 907	AX-111103882 (A)	6B	123.7	121.5-123.9	0.15	4.9E-3	0.005	11.0	FarmCPU	Stay-green [20]
**Low N**										
27.5.20 777	AX-158578529 (C)	3B	710.4	710.4-718.7	0.20	1.3E-3	−0.006	32.4	BLINK	--
9.6.20 907	AX-111561744 (G)	2D	23.4	21.9–23.4	0.05	4.6E-5	−0.016	63.8	BLINK	See above
24.6.20 1107	BS00067216_51 (C)	3 A	83.7	74.2–83.7	0.07	8.7E-5	0.018	36.0	BLINK	--
24.6.20 1107	Tdurum_contig30082_197 (A)	6 A	109.4	93.8-230.7	0.47	2.5E-2	−0.008	10.0	FarmCPU	--

*SNP* Single nucleotide polymorphism, *Chr* Chromosome, *Pos (Mbp)* Position of the SNP on the chromosome in mega base pairs, *Haplotype (Mbp)* Range of the haplotype in Mbp (Tab. S6a-l), *MAF* Minor allele frequency, *B&H p-value* Benjamini-Hochberg p-value, *Effect* Magnitude of estimated marker effect from GWAS model, *PVE* Percentage of phenotypic variance explained (a function of magnitude of marker effects and MAF), *Model* Utilised GWAS model, *Prev. reported* SNP or haplotype has been reported in previous studies or identified in the reference year 2019, *NAB* Nitrogen in aboveground plant biomass, *NUEbio* Nitrogen use efficiency for biomass production, *NUpE* Nitrogen uptake efficiency, *PBW* Plant biomass weight, *SDW* Shoot dry weight, *SPAD* Chlorophyll content, *YII* Effective photochemical quantum yield of PS II

Under low N availability, four large effect loci were significantly associated with stay-green (Table 2). One of them had also been found across N levels (AX-111561744). Three of these large effect loci were exclusively identified under low N availability (AX-158578529, BS00067216_51 and Tdurum_contig30082_197). Of these, BS00067216_51 and AX-158578529 explained the highest proportions of phenotypic variance accounting for 36.0 and 32.4%, respectively. The maker BS00067216_51 has a minor allele with favourable effects on stay-green. The MTAs identified under low N availability in 2020, however, did not match those identified in the reference year 2019.

### Identified haplotypes enhance stay-green performance and grain yield

**Fig. 9:**
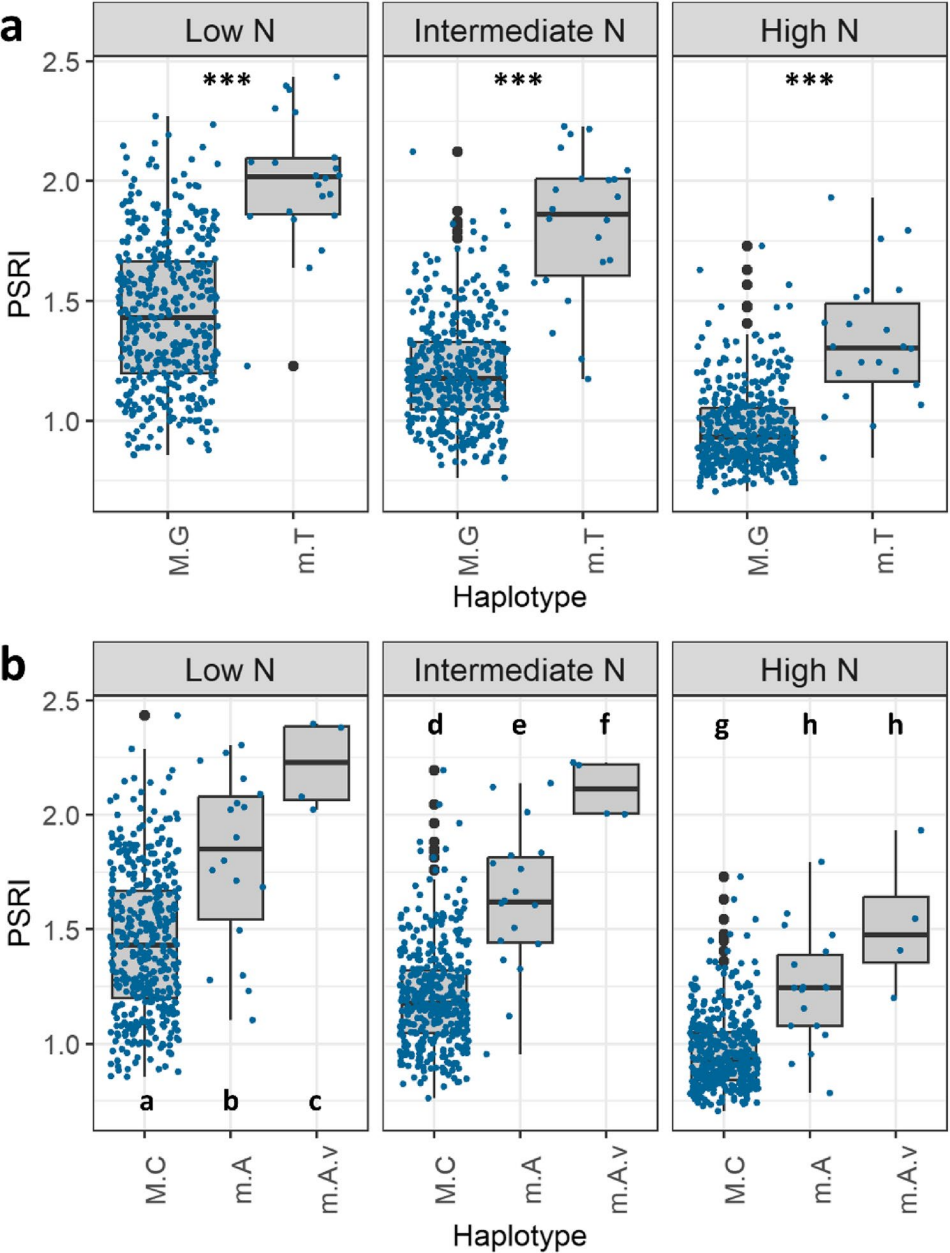
The stay-green indicator PSRI for the two favourable major allele haplotypes of the markers (**a**) AX-111561744 and (**b**) AX-158618766. Haplotype coding: *M* Major allele, *m* Minor allele, *G* Guanine, *C* Cytosine, *T* Thymine, *A* Adenine, *v* Variant thereof. Imaging date: 9 June 2020 (907 GDDs). Significance coding: different letters indicate significant differences. *** p≤0.001

Haplotype analyses revealed that the favourable major allele haplotypes identified for the markers AX-111561744 and AX-158618766 significantly enhance stay-green and grain yield at all studied N levels (Fig. 9, Fig. S2). The favourable variants of these two haplotypes were present in 95% of the stay-green cultivars, i.e. the highest performing 25% of cultivars in the diversity set based on PSRI values during the central senescence phase (9 and 24 June 2020).

The two favourable major allele haplotypes additionally identified under low N availability (AX-158578529 and Tdurum_contig30082_197) also showed an increasing effect on stay-green and grain yield. Significance of the effects was dependent on the respective haplotype variants since there were multiple for these SNP markers.

**Fig. 10:**
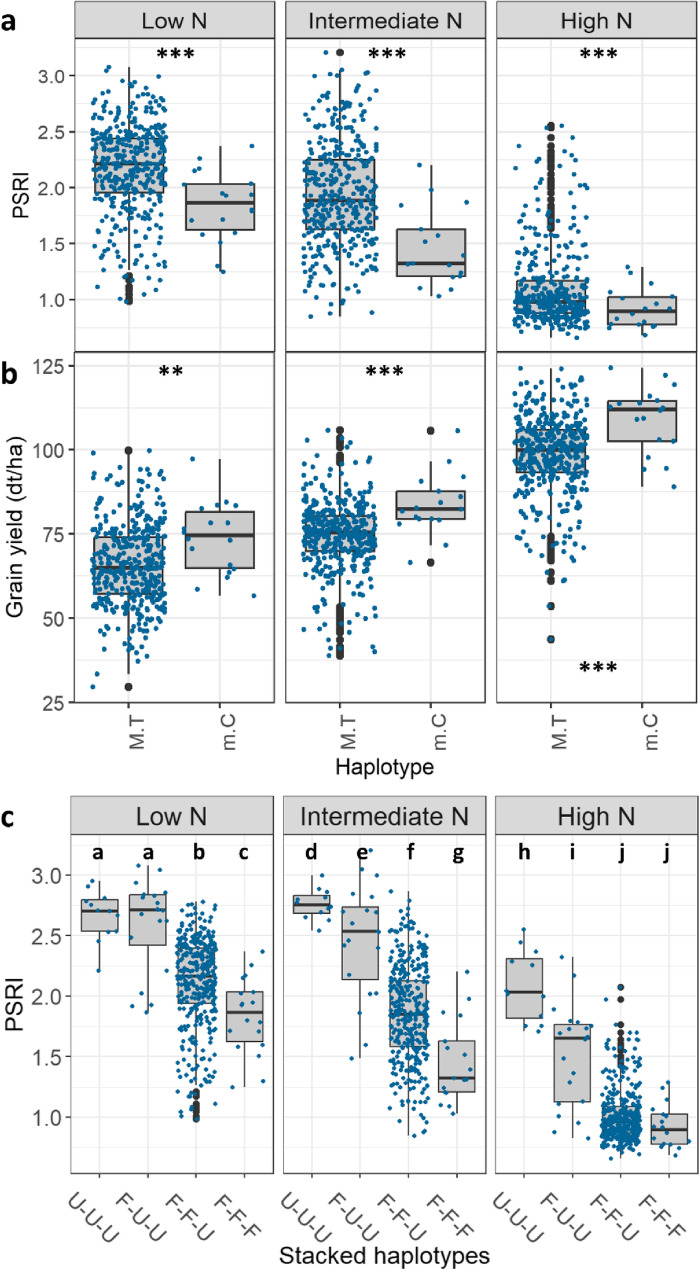
(**a**) The stay-green indicator PSRI and (**b**) grain yield by haplotypes of the marker BS00067216_51 and N level. Haplotype coding: M = major allele, m = minor allele, C = cytosine, T = thymine. **c** PSRI by N level and different combinations of the favourable haplotype variants of the markers AX-111561744, AX-158618766 and BS00067216_51. U-U-U = unfavourable variants of all three haplotypes; F-U-U = one favourable haplotype variant (AX-111561744); F-F-U = two favourable haplotype variants (AX-111561744 and AX-158618766) and F-F-F = three favourable haplotype variants combined in the same cultivars (BS00067216_51, AX-111561744 and AX-158618766). Imaging date: 24 June 2020 (1107 GDDs). Significance coding: different letters indicate significant differences. *** p≤0.001 and ** p≤0.01

Among the two large effect loci with favourable minor alleles, the haplotypes of the marker BS00067216_51 identified under low N availability showed the most significant differences. Cultivars carrying the favourable haplotype variant showed prolonged stay-green and elevated grain yields at all studied N levels (Fig. 10a/b). Hence, the stay-green enforcement by the favourable haplotype variant of the marker BS00067216_51 was consistent and functional. When the favourable minor allele haplotype of the marker BS00067216_51 was stacked onto those of the markers AX-111561744 and AX-158618766, it further increased stay-green at low and intermediate N availability (Fig. 10c).

### Allele frequencies over time reveal indirect selection for stay-green

Based on the present diversity set, both of the large effect loci with favourable minor alleles (BS00067216_51 and AX-111103882) show an increase in favourable allele frequency over the past decades. The favourable allele of the marker AX-111103882 increased from around 5% in the 1980 s to 24% in the 2010 s; whereas the favourable allele of BS00067216_51 first occurred in German cultivars between the year 2000 and 2009 and increased up to 10% in the following decade. In addition, Tdurum_contig30082_197 showed an increase from 25% favourable allele frequency in the 1960 s to 70% in the 2010 s turning from a minor into a major allele among the German cultivars in this panel.

## Discussion

In this study, 221 wheat cultivars were grown under three different soil N levels to characterise the stay-green behaviour of cultivars and find responsible genetic regions. This was done using a combination of UAV-based multispectral image analyses over time and genome-wide association studies followed by haplotype analyses.

The plant senescence reflectance index was identified as the most specific stay-green indicator, allowing for differentiation of genotypic effects due to its higher sensitivity to senescence-related changes in pigment composition and its higher reliability. We found genetic variance for stay-green and a consistent genetic correlation between stay-green and grain yield at all imaging dates and N levels within the utilized diversity panel, highlighting its potential as a future breeding target. Haplotype analyses revealed two favourable major allele haplotypes present in 95% of the stay-green cultivars, which significantly delay senescence and increase grain yield. In addition, we identified a favourable minor allele haplotype specifically associated with stay-green under low N availability and capable of further increasing stay-green and grain yield when stacked onto the two favourable major allele haplotypes. Relative frequencies of favourable minor alleles at several large effect loci showed an increase in the German cultivars over the past decades indicating that there may have been an indirect selection for stay-green alleles in the course of wheat breeding.

### PSRI identified as the most sensitive stay-green indicator

We have examined the three vegetation indices NDVI, NDRE and PSRI for their suitability as stay-green indicators. The first two are classical vegetation and biomass indices used in remote sensing and the latter one has been developed to assess ageing processes at the leaf and fruit level. All three of them have been used in recent studies to track senescence in cereals at the canopy level [20, 31, 57].

In the regression analysis, canopy level NDVI was able to predict the chlorophyll content determined at the leaf scale. However, the saturation effect in the NDVI data seriously limits its sensitivity and precision in senescence tracking. Saturation is a known phenomenon with NDVI from closed canopies [78]. In addition, NDVI has been reported to be insensitive to physiological changes occurring during early senescence at the leaf scale [36, 79]. NDRE is a common alternative to NDVI as it overcomes the saturation issues and offers a deeper canopy penetration and a higher sensitivity to changes in chlorophyll content [57, 80]. This is primarily an effect of replacing the reflectance in the red by reflectance in the red-edge spectral region, which is less prone to saturation at high chlorophyll contents at the leaf and canopy scale and more robust to variation in vegetation structure [57, 80]. However, the PSRI was reported to be more sensitive to changes in leaf reflectance occurring specifically during senescence [31, 36]. The index incorporates a red/blue ratio allowing to detect changes in the relative contents of chlorophylls to carotenoids taking place in the course of senescence which is supposed to make the PSRI more sensitive to senescence-specific changes [35]. Although the PSRI was developed to track senescence or ripening processes at the leaf and fruit scale, several studies have proven its suitability for UAV-based senescence phenotyping at the canopy level [81, 82].

In addition, reliability according to Bernardo [60] was constantly and considerably higher for PSRI than for NDRE (Fig. 5). Since reliability is an important criterion for the analysis of genotypic effects, only PSRI was considered a suitable trait for GWAS. We have therefore identified the PSRI as the most informative stay-green indicator among the vegetation indices evaluated in this study. Its greater sensitivity to changes in pigment composition and its higher reliability make it a valuable tool for studying senescence dynamics, especially in experiments on large diversity panels requiring accurate differentiation between genotypic effects.

In the relationship between PSRI and SPAD, it is striking that the genetic correlation was lower than the correlation across N levels and the strength of the correlation was dependent on the respective N level (Fig. 2). Under low N availability, genotypic differences in chlorophyll content were larger and the correlation between chlorophyll content and PSRI was stronger. This suggests that under low N conditions the PSRI was more strongly determined by chlorophyll content, whereas at high N supply other genotypic differences such as pigment composition, leaf structure and canopy architecture weakened the genetic correlation between PSRI and SPAD. This is in line with expectations due to different measurement levels (canopy versus leaf level) and wavelengths used for determination of PSRI and SPAD. Moreover, all optical measurements have a limited linear range and there may be slight signs of saturation at the lower end of the value range of PSRI under high N availability.

### High NUE can enhance stay-green performance under low N availability

Stay-green cultivars showed not only a lower starting PSRI level but also lower relative senescence rates (Fig. 4b), which could even result in a temporary stagnation of the process during the central senescence phase between 9 and 24 June 2020. Thus, senescence can show a non-linear course [57] and does not necessarily resemble a sigmoidal curve as often assumed by modellers [33, 34]. This stay-green behaviour was more pronounced at high than at low N availability which is consistent with other studies [21, 83]. The observed N effect is also in line with the senescence model by Borrell [23] assuming that stay-green is the result of the source-sink balance between N requirement by the grain (determined by grain number) and N availability (N uptake and senescence-induced N remobilisation processes) in the grain filling period when water and nutrients become scarce [24]. Grain N is for the most part fuelled by remobilisation of N taken up before flowering and stored in leaves, shoot and roots [83, 84]. Hence, a high N uptake and utilisation efficiency especially before but also after flowering can enhance stay-green performance and grain filling [25], particularly under low N availability [84]. This is also reflected by the higher correlation of NUE and grain yield with stay-green under low N availability in comparison with higher N levels (Fig. 6). Our results are in line with the common understanding that prolonged leaf greenness is associated with maintenance of photosynthetic activity and increased translocation of assimilates to harvested tissues and is therefore positively correlated with grain yield [12, 13]. However, this relationship is complex and there are studies reporting low or no correlations between stay-green performance and grain yield when other factors are limiting yield formation such as low grain number [85]. A low sink strength in the source-sink balance between N supply and N demand can allow the plant to stay green while at the same time limiting grain yield [24, 85]. A high stay-green capacity is also not favourable when it is accompanied by a low N uptake efficiency and low N storage leading to a low grain protein content and consequently poor wheat quality [86]. This shows that grain yield and grain quality are complex polygenic traits in cereal crops and the underlying processes need to be finely tuned to ensure yield quantity and quality. Grain yield in cereals can be seen as the result of source-related and sink-related traits. Source-related traits are traits underlying light interception and radiation use efficiency, e.g. root and canopy structure [87, 88] or Calvin cycle efficiency, while sink-related traits are traits determining grain set and grain size, e.g. spike fertility and carbohydrate storage and remobilisation [88]. Stay-green capacity is a source-related trait forming part in this complex source-sink network that together with environmental factors ultimately determines yield in wheat [88]. This source-sink concept originally refers to photosynthetic assimilation, but may be extended to include N metabolism.

### Favourable minor allele haplotype increases stay-green and yield at all N levels

The favourable minor allele marker BS00067216_51 is particularly interesting due to its significant association with stay-green under low N availability and the increasing effects of its favourable haplotype variant on stay-green and grain yield at all studied N levels (Fig. 10a + b). The two favourable major allele markers explaining the largest proportions of stay-green variance across N levels and showing enhancing effects on stay-green and grain yield in the haplotype analyses (AX-111561744 and AX-158618766), were found to be present in 95% of the stay-green cultivars in the panel (Fig. 9, Fig. S2). When the favourable minor allele haplotype of BS00067216_51 was stacked onto these two favourable major allele haplotypes in the same cultivar, it significantly added to their stay-green effects (Fig. 10c). This additive effect was only significant under low and intermediate N availability, revealing G×E interactions between individual haplotype variants and N levels. In the mixed model ANOVA, the overall interactive effect between cultivar and N level on PSRI was not significant. However, this is not a contradiction, as interactive effects between individual treatment levels and haplotype variants are very diverse and not necessarily significant at the panel level. The observed G×E interactions support our understanding that the stay-green phenotype relies on a combination of different underlying genes and traits, which may vary depending on the respective environment. The additive stay-green effect of the favourable haplotype variant of BS00067216_51 along with its specificity for low N availability makes it an interesting candidate for crossing experiments. This is particularly true, as its favourable haplotype variant seems to be relatively new in the German wheat breeding germplasm having first occurred between the years 2000 and 2009.

SNP markers within the haplotype block of AX-111561744 have previously been associated with chlorophyll content, photosynthetic efficiency, biomass, N in aboveground biomass, NUE for biomass production and N uptake efficiency [77]. The finding that a genetic marker associated with stay-green across N levels coincides with genetic regions previously identified for NUE-related traits, highlights the major role of nitrogen in senescence regulation and stay-green performance not only when N is limiting.

### Allele frequencies imply indirect selection for stay-green alleles over the past decades

Based on the present diversity panel, both favourable minor alleles corresponding to large effect loci (BS00067216_51 and AX-111103882) increased in frequency since their first occurrence in the German wheat breeding germplasm. In addition, the favourable allele at the large effect locus of the marker Tdurum_contig30082_197 even turned from a minor into a major allele over the past 60 years. These results suggest that favourable alleles shaping the stay-green behaviour of modern German wheat cultivars were introduced and possibly indirectly selected for over the past decades. This probably happened by selecting for higher and more stable yields. However, the conclusions drawn here are only based on the utilised diversity panel and may not reflect the whole picture.

### Limitations

One of the most holistic ways of characterising genotypic senescence development is to model senescence kinetics and quantify total plant greenness from heading to maturity [57]. However, due to the sensitivity of image acquisition methods to weather conditions, high quality image data were not available in sufficiently high temporal resolution for the crucial period to model cultivar-specific senescence development. As an approximation, we therefore estimated the genotypic effects on the stay-green indicator PSRI across the central senescence period (9 and 24 June 2020).

Since total plant greenness from heading to maturity could not be determined, the question arose as to whether the cultivar effects needed to be corrected for individual deviations in heading dates. However, heading date does not immediately determine maturity date with a correlation ranging from *r* = 0.75 to *r* = 0.86 based on a study on adapted sets of cultivars [89]. Moreover, in the present diversity set maturity groups are largely overlapping with the population structure arising from different eras of plant breeding in different geographic locations. However, the population structure has already been accounted for in the GWAS by mixed-method modelling and the use of principal components as covariates. Since a double correction must be avoided [90], no additional correction for heading date was performed in this study. Regular rainfall and the absence of pronounced heat periods throughout the entire data collection phase did not make any adjustments due to weather conditions necessary.

Of the six large effect loci identified by the GWAS on the 2020 dataset, two (33%) had already been found in the reference year 2019. The limited overlap was to be expected because not only consecutive years but also swapping fields, shifting imaging dates relative to plant development, slightly differing N concentrations at the low N level, and minor deviations in agricultural management procedures create a different environment for the plants. In addition, different camera systems were used (MicaSense RedEdge Dual in 2020 and MicaSense RedEdge-MX in 2019) and therefore the PSRI had to be calculated using different NIR bands in 2020 (740 nm) and 2019 (842 nm).

### Relevance of the presented results for future studies

We have identified favourable major allele haplotypes present in 95% of stay-green cultivars as well as a favourable minor allele haplotype that was shown to further improve stay-green performance when the favourable haplotype variants were combined in a cultivar. The additive behaviour of haplotype effects is in line with our understanding of stay-green being under complex genetic control [20, 85, 91]. In previous studies, genetic regions identified for stay-green have been shown to coincide with loci associated with phenology, growth, plant height, and yield [19, 37]. A gene ontology enrichment analysis conducted on candidate genes located within stay-green quantitative trait loci (QTL) in wheat indicated an association of these genes with biological processes such as leaf senescence, ethylene response, and apoptosis and with functions mainly related to nutrient reservoir activity [20]. Furthermore, a comparative transcriptome analysis has shown that genes for leaf senescence, photosynthesis, chlorophyll metabolism and antioxidative enzyme activity were upregulated during leaf senescence in the stay-green wheat cultivars compared to non-stay-green cultivars [92]. These results imply that a stay-green phenotype arises from a large number of underlying genes and traits acting at different levels and time points during the senescence process [19]. This can be exploited by stacking multiple favourable alleles in marker-assisted crossing experiments. The stay-green haplotypes identified in this study can serve as a basis for stacking favourable minor allele haplotypes onto favourable major allele haplotypes.

The G×E interactions observed in this study imply that some of the identified genetic regions may convey a specific benefit under certain environmental conditions whereas others may have a robust effect across different environments [19]. Therefore, a more differentiated understanding of the genetic and physiological basis of stay-green is required to ascertain which genetic regions can further improve stay-green performance across different environments or under specific environmental conditions. With regard to N availability, the newly identified stay-green haplotypes can contribute to a more in-depth understanding by guiding the identification of the underlying genes.

## Conclusions

By combining multispectral image analyses with genomic tools, we identified a favourable minor allele haplotype with large effect specifically associated with stay-green under low N availability. Cultivars carrying the favourable haplotype variant showed significantly higher stay-green capacities and grain yields at all studied N levels. In addition, our study identified two favourable major allele haplotypes present in the majority (95%) of stay-green cultivars. When all three haplotypes are combined in a cultivar, the favourable minor allele haplotype significantly adds to the stay-green effects of the favourable major allele haplotypes. This additive effect was only significant under low and intermediate N availability. These findings support our understanding that the stay-green phenotype arises from a combination of multiple underlying genes and traits, which may vary depending on the respective environment. Our results confirm that stay-green may be a suitable breeding target to develop high-yielding wheat cultivars that are either specifically adapted to certain conditions (e.g. low N availability) or stable across a range of environments. The presented relative allele frequencies suggest that the stay-green trait has already been indirectly selected for during the past decades of wheat breeding. Novel wheat cultivars with enhanced stay-green performance due to a high NUE have the potential to contribute to security as well as to sustainability of wheat production by ensuring yields in low-input systems and allowing for a reduction in N fertilisation in high input systems.

Our results show that the utilised UAV-based multispectral phenotyping method is capable of precisely quantifying and differentiating genotypic effects on stay-green in a large diversity panel under different treatment conditions over time. The choice of a sensitive and reliable stay-green indicator was shown to be of particular importance. The utilised combination of image analyses and GWAS is a highly dynamic field and particularly the use of deep learning approaches has the potential to further promote crucial advances in dynamic digital phenotyping [93]. 

## Supplementary Information

Supplementary Material 1.

Supplementary Material 2.

Supplementary Material 3.

## Data Availability

The datasets generated and/or analysed in this study are available in the Jülich DATA repository:10.26165/JUELICH-DATA/FSQODY.
