# Correction: UAV-based multispectral image analysis revealed stay-green haplotypes in wheat specific for different soil nitrogen levels

**DOI:** 10.1186/s12870-025-07791-1

**Published:** 2025-11-24

**Authors:** Helen Behn, Agim Ballvora, Juliane Bendig, Facundo R. Ispizua Yamati, Ahossi Patrice Koua, Anne-Katrin Mahlein, Annaliese S. Mason, Uwe Rascher, Mohammad Bahman Sadeqi, Jens Léon

**Affiliations:** 1 Plant Breeding Department, Institute of Crop Science and Resource Conservation, University of Bonn, Kirschallee 1, Bonn, 53115 Germany; 2 Faculty of Agricultural, Nutritional and Engineering Sciences, University of Bonn, Campus Klein-Altendorf, Klein-Altendorf 2, Rheinbach, 53359 Germany; 3 Institute of Sugar Beet Research (IfZ), Holtenser Landstraße 77, Göttingen, 37079 Germany; 4 Institute of Bio- and Geosciences, IBG-2: Plant Sciences, Forschungszentrum Jülich GmbH, Leo-Brandt-Straße, Jülich, 52425 Germany; 5 Institute of Crop Science and Resource Conservation, Faculty of Agricultural, Nutritional and Engineering Sciences, University of Bonn, Bonn, 53115 Germany

**Correction: BMC Plant Biol 25, 1405 (2025)**

**https://doi.org/10.1186/s12870-025-07441-6**

Following publication of the original article [1], the authors found errors in the reference citations in the body. The errors had been introduced during the layout process. These errors are now being corrected.

The corrections do not affect the conclusion of the article. The original article [1] has been corrected.
