# Supplementary material for: UAV-based multispectral image analysis revealed stay-green haplotypes in wheat specific for different soil nitrogen levels
Source: BMC Plant Biol. 2025 Oct 21;25:1405. doi: 10.1186/s12870-025-07441-6 (PMC12539009; doi:10.1186/s12870-025-07441-6)
Supplement: Supplementary file 2 — Supplementary Material 2. [file 12870_2025_7441_MOESM2_ESM.docx]

**Tab. S7:** Summary of PSRI and NDRE mean values, minima (Min), maxima (Max), estimates of reliability according to Bernardo (59) with standard error (SE) and coefficients of variation (CV) for all four imaging dates and N levels. The significance of the effects of N level (N), cultivar (C), growing degree days (GDDs) and their interactions is displayed as the F statistic and the p-value from the mixed model ANOVA. The given p-values were calculated using the Kenward-Roger method and subsequently subjected to a Greenhouse-Geisser correction.

| **Imaging dates (GDDs/date)** | **N Level**  **(kg/ha)** | **PSRI** | | | | **NDRE** | | | |
| --- | --- | --- | --- | --- | --- | --- | --- | --- | --- |
|  |  | **Mean** | **Min - Max** | **Reliability**  **(SE)** | **CV** | **Mean** | **Min - Max** | **CV** | **Reliability**  **(SE)** |
| 777  27 May 2020 | 81 | 1.21 | 0.03 - 2.40 | 0.15  (0.039) | 40.10 | 0.48 | 0.25 - 0.69 | 19.18 | 0.13  (0.038) |
|  | 110 | 0.75 | -0.17 - 1.84 |  | 46.41 | 0.56 | 0.29 - 0.73 | 11.63 |  |
|  | 220 | 0.18 | -0.27 - 1.52 |  | 125.77 | 0.70 | 0.37 - 0.81 | 8.65 |  |
| 907  9 June 2020 | 81 | 1.47 | 0.86 - 2.44 | 0.50  (0.035) | 22.54 | 0.38 | 0.16 - 0.58 | 21.79 | 0.30  (0.037) |
|  | 110 | 1.24 | 0.76 - 2.23 |  | 21.10 | 0.45 | 0.20 - 0.62 | 14.66 |  |
|  | 220 | 0.99 | 0.71 - 1.93 |  | 19.05 | 0.60 | 0.32 - 0.68 | 9.30 |  |
| 1107  24 June 2020 | 81 | 2.15 | 0.98 - 3.08 | 0.55  (0.024) | 18.71 | 0.21 | 0.08 - 0.49 | 38.56 | 0.40  (0.035) |
|  | 110 | 1.91 | 0.85 - 3.21 |  | 24.33 | 0.27 | 0.10 - 0.53 | 31.07 |  |
|  | 220 | 1.09 | 0.66 - 2.55 |  | 31.55 | 0.51 | 0.21 - 0.64 | 16.38 |  |
| 1290  7 July 2020 | 81 | 2.43 | 1.76 - 3.16 | 0.58  (0.023) | 8.42 | 0.13 | 0.09 - 0.19 | 11.96 | 0.23  (0.039) |
|  | 110 | 2.45 | 1.76 - 3.33 |  | 9.48 | 0.14 | 0.10 - 0.19 | 11.39 |  |
|  | 220 | 2.36 | 1.25 - 3.51 |  | 13.52 | 0.19 | 0.12 - 0.40 | 20.87 |  |
| Significance of effects  (F statistic/p-value) | N | 15.45 * / 0.029 | | | | 28.37 * / 0.013 | | | |
|  | C | 8.54 *** / <0.001 | | | | 6.10 *** / <0.001 | | | |
|  | N*C | 0.78 / 0.990 | | | | 0.90 / 0.841 | | | |
|  | GDDs | 11454.45 *** / <0.001 | | | | 69127.11 *** / <0.001 | | | |
|  | GDDs*N | 35.68 *** / <0.001 | | | | 2452.48 *** / <0.001 | | | |
|  | GDDs*C | 4.17 *** / <0.001 | | | | 8.73 *** / <0.001 | | | |
|  | GDDs*N*C | 0.87 / 0.916 | | | | 1.91 *** / <0.001 | | | |

**Tab. S8:** Summary of relative senescence rates, i.e. average percentage change in PSRI value, from 9 June to 24 June 2020 (907 to 1107 GDDs) giving means and standard deviations (SD) for the complete diversity panel and the ten highest and lowest performing cultivars based on their average genotypic effects on the Plant Senescence Reflectance Index (PSRI). The ten highest performing cultivars were Avenir, Pionier, Vuka, Discus, Boxer, Gourmet, Robigous, Matrix, Mentor and Julius. The ten cultivars with the poorest stay-green performance were NS-46-90, Pobeda, Cajeme 71, Ivanka, Renesansa, Siete Cerros, Triple dirk S, NS-66-92, Benni multifloret and Centurk. Reliability of the relative senescence rate refers to the complete set of cultivars and the time period between 9 June and 24 June 2020.

| **Cultivars** | **Statistic** | **Relative senescence rate** | | |
| --- | --- | --- | --- | --- |
|  |  | **Low N** | **Intermediate N** | **High N** |
| **Complete diversity panel** | Mean | 48.9 | 54.7 | 9.4 |
|  | SD | ±18.0 | ±24.9 | ±18.3 |
| **High performers** | Mean | 30.9 | 28.0 | -4.9 |
|  | SD | ±8.9 | ±10.7 | ±4.5 |
| **Low performers** | Mean | 33.3 | 47.0 | 56.6 |
|  | SD | ±14.4 | ±20.0 | ±24.9 |
| **Reliability** |  | 0.24 | | |
