# Supplementary material for: UAV-based multispectral image analysis revealed stay-green haplotypes in wheat specific for different soil nitrogen levels
Source: BMC Plant Biol. 2025 Oct 21;25:1405. doi: 10.1186/s12870-025-07441-6 (PMC12539009; doi:10.1186/s12870-025-07441-6)

**Fig. S1:** Pearson correlation between PSRI on 7 June 2019 (808 GDDs) and 9 June 2020 (907 GDDs) to compare stay-green performance of individual cultivars between different years. R = Pearson correlation coefficient, p = p-value.


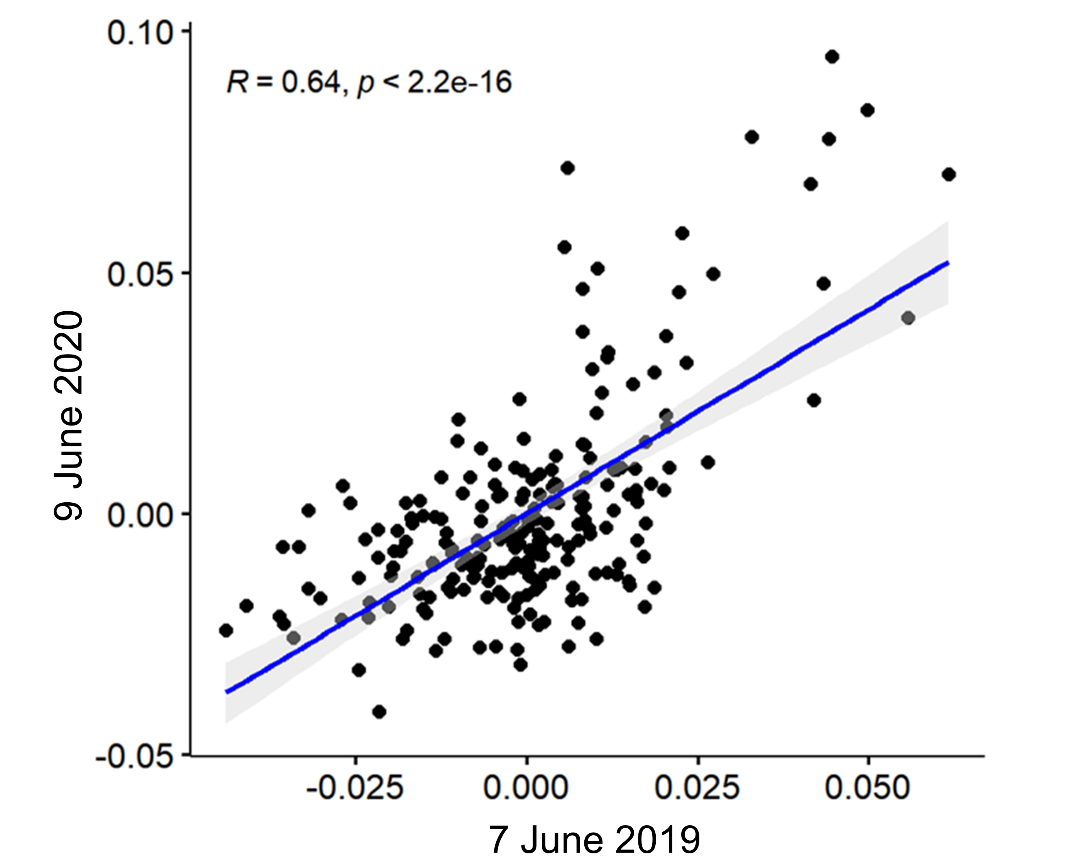


**Fig S2:** Grain yield by haplotypes of the markers a) AX-111561744 and b) AX-158618766 to estimate functionality of stay-green enhancement. Significant differences are indicated by asterisks in case of two groups and by different letters in case of more than two groups. The significance level of the indicated differences was at least α = 0.05. Haplotype coding: M = major allele, m = minor allele at marker position, C = cytosine, T = thymine, A = adenine, G = guanine, v = variant thereof.


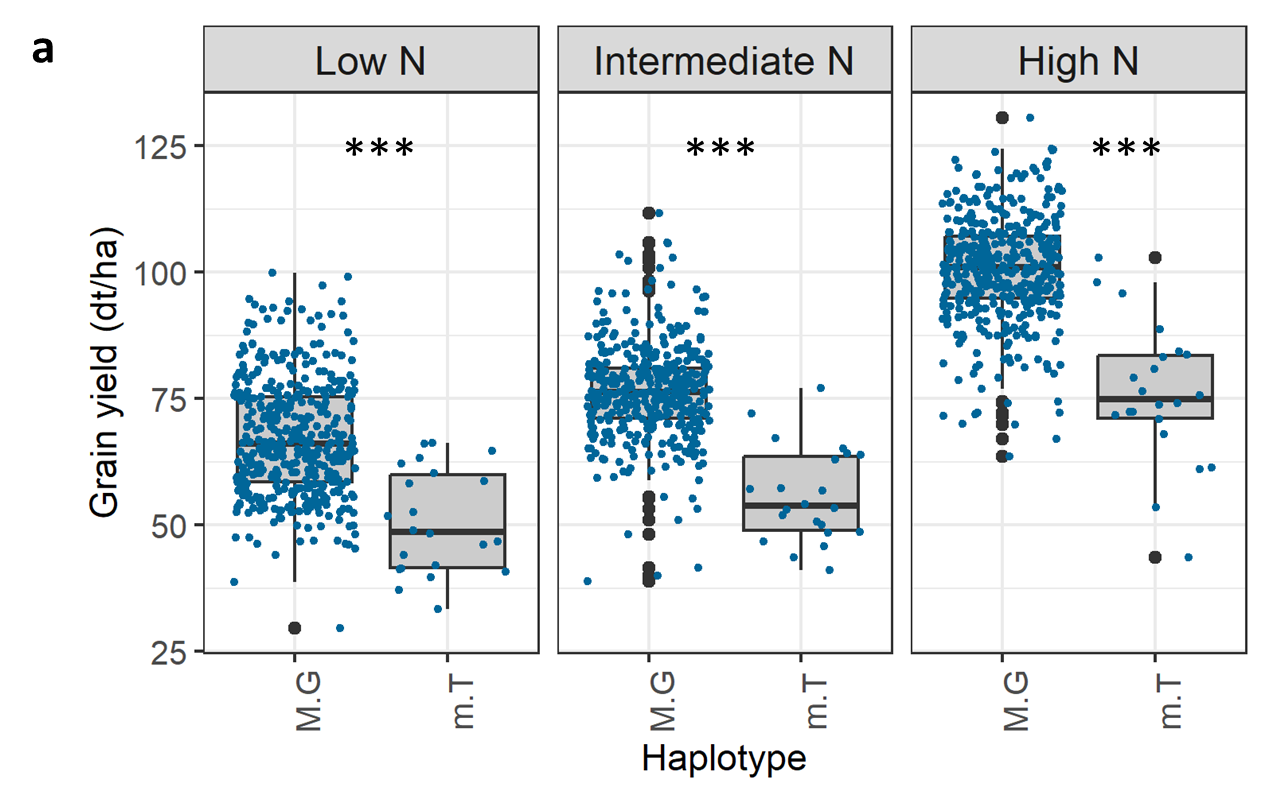


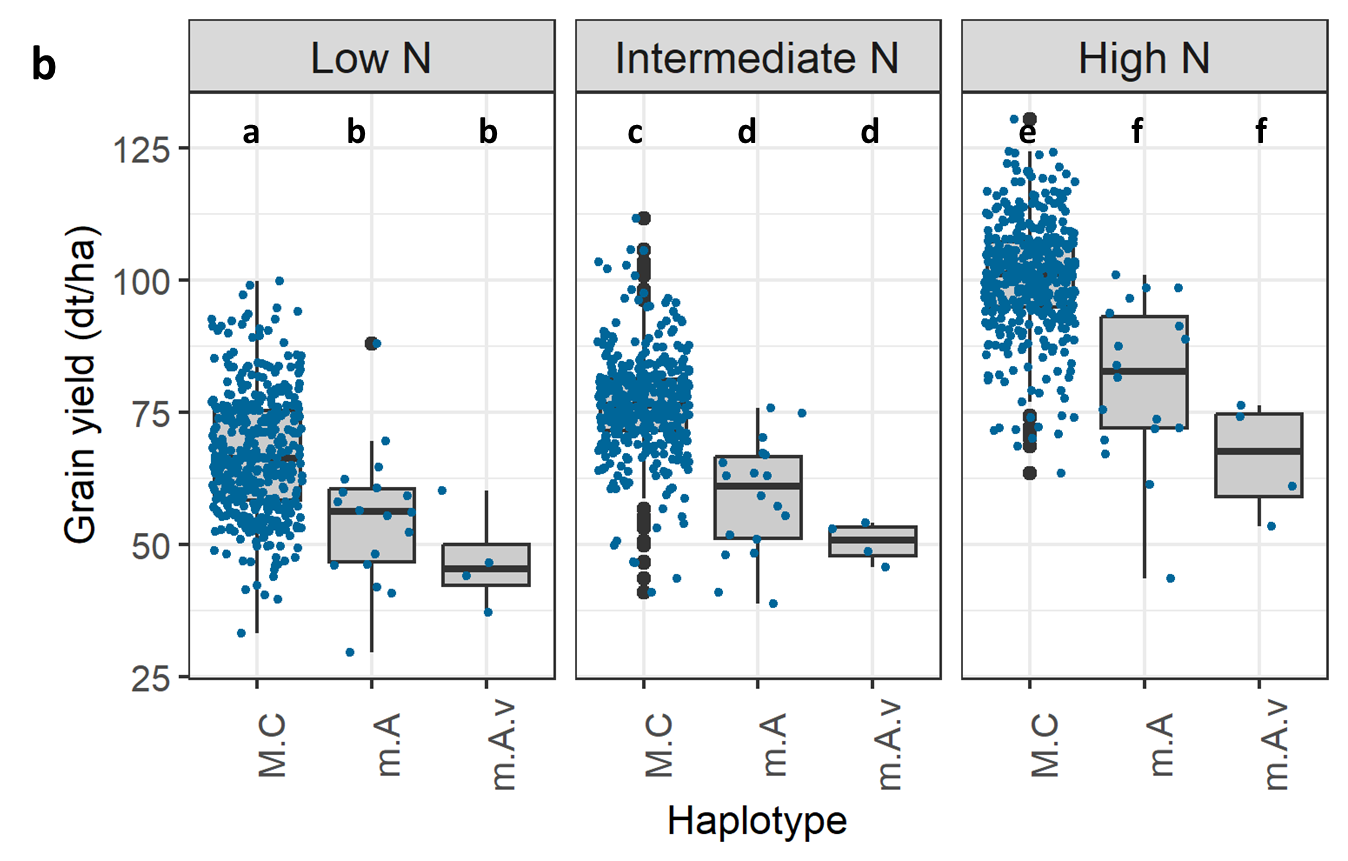

Supplement: Supplementary file 3 — Supplementary Material 3. [file 12870_2025_7441_MOESM3_ESM.docx]
